# Pituitary Hyperplasia in Severe Primary Hypothyroidism: A Case Report and Review of the Literature

**DOI:** 10.1155/2019/2012546

**Published:** 2019-06-25

**Authors:** Prateek Shukla, Ketan R. Bulsara, Pooja Luthra

**Affiliations:** ^1^Division of Internal Medicine, UConn Health Center, Farmington, CT 06030, USA; ^2^University of Connecticut School of Medicine, Farmington, CT 06030, USA; ^3^Division of Neurosurgery, UConn Health Center, Farmington, CT 06030, USA; ^4^Division of Endocrinology and Metabolism, UConn Health Center, Farmington, CT 06030, USA

## Abstract

Pituitary hyperplasia is commonly present but remains largely undiagnosed in primary hypothyroidism. It is easily reversible with thyroid replacement therapy. If imaging is performed prior to biochemical evaluation, then patients may undergo pituitary surgery. We present the case of a 34-year-old female with profound primary hypothyroidism and secondary pituitary hyperplasia that resolved after thyroid hormone supplementation. We will discuss the current literature regarding pituitary hyperplasia in primary hypothyroidism in adults.

## 1. Introduction

Pituitary hyperplasia is a relatively common condition that occurs in both physiological and pathological states. Pregnancy is the most common condition associated with physiological pituitary enlargement, associated primarily with lactotroph hyperplasia [1, 2]. Pathological hyperplasia has been shown to be associated with end organ insufficiency from primary gonadal insufficiency [3, 4], primary adrenal insufficiency [5], and primary hypothyroidism [2, 6–50]. Although traditionally thought to be uncommon [44], pituitary enlargement is common in primary hypothyroidism and has now been well documented in the literature [2, 6–50].

We present an interesting case of a 34-year-old female with no prior diagnosis of hypothyroidism but a long history of hypothyroid symptoms, who presented with sudden onset of neurologic symptoms and an enlarged pituitary gland secondary to profound primary hypothyroidism. We will also review the published literature on pituitary hyperplasia secondary to primary hypothyroidism.

## 2. Case Report

**Figure 1:**
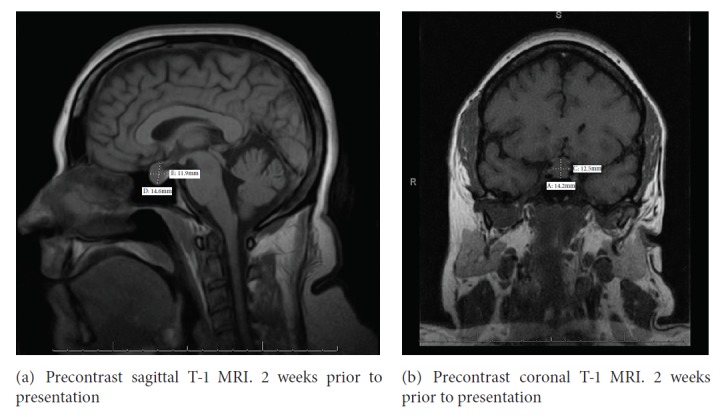


Our patient is a 34-year-old Caucasian female with a past history of attention deficit hyperactivity disorder, hypertension, irritable bowel syndrome, and anxiety disorder who had a long standing history of dry skin and abnormal menstrual periods. She is nulligravida and had menorrhagia with irregular menses and increasing polymenorrhea, several months prior to presentation. She also had a history of chronic diarrhea alternating with constipation but had experienced a recent 20 lb weight gain. 1 month prior to presentation, she developed galactorrhea with breast enlargement and tenderness. 3 weeks prior to presentation, evaluation by her gynecologist revealed a thyroid stimulating hormone (TSH) >150 mIU/mL (0.5-5.5) free thyroxine (FT4) 0.4 ng/dL (0.61-1.82) and prolactin 29.4 ng/mL (3.34-26.72). An MRI of the pituitary at an outside facility revealed an enlarged pituitary gland measuring 14x12 mm, abutting the optic chiasm (Figures 1(a) and 1(b)). She presented to the hospital with a 1-day history of transient blurry vision, apraxia and aphasia, transient chest tightness, and increasing anxiety. The vision changes were described as an inability to focus on near objects with no reported loss of visual fields. She denied any pain or swelling in her neck, recent viral infections, excessive iodine intake, exposure to iodinated contrast agents, lithium or amiodarone, and any history of prolonged steroid use. She denied any increasing skin tags, changes in appearance or changes in ring or shoe size. There were no symptoms suggestive of adrenal insufficiency. Her medications included amphetamine, nebivolol, clonazepam, citalopram, hydrochlorothiazide, polyethylene glycol, and levocetirizine. Family history was significant for hyperthyroidism in her sister but was otherwise unremarkable for any pituitary disorders.

**Figure 2:**
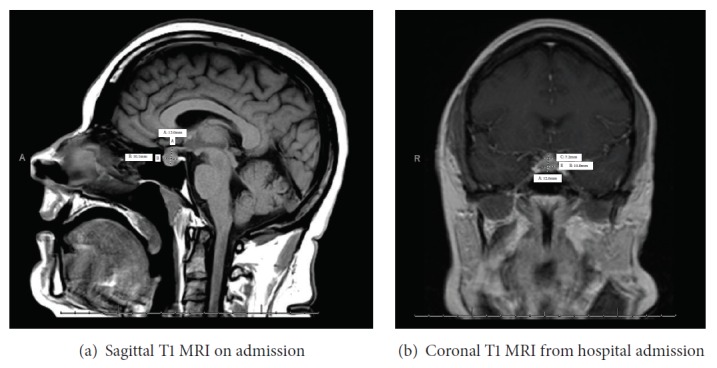


On physical exam, she had a body mass index of 41.53 kg/m^2^, blood pressure of 110/89 mmHg, heart rate being regular at 62 beats per minute, and breathing 14 breaths per minute with oxygen saturations of 96% on ambient air. She was obese and in no distress, and oriented to time, place, and person. There was no obvious thyromegaly. No abdominal stria was noted and overall exam was unremarkable other than dry skin. Her neurological exam was normal and there were no obvious visual field deficits on bedside examination. Laboratory evaluation showed a (Table 1) TSH of 251.21 mIU/mL (0.5-5.5), total T4 of 2.07 *μ*g/dL (4.87-11.72), FT4 of 0.44 ng/dL (0.61-1.82), free triiodothyronin (FT3) of 1.5 pg/mL (2.2-4.2), prolactin of 29.48 ng/mL (3.34-26.72) (23.5 ng/ml by dilution), estradiol 20 pg/mL, Luteinizing Hormone (LH) 2.52 mIU/mL, Follicle Stimulating Hormone (FSH) 5.03 mIU/mL, morning cortisol 8 *μ*g/dL, adrenocorticotrophic hormone (ACTH) 10 pg/mL (6-58), insulin-like growth factor-1 (IGF-1) 109 ng/mL (108-368), Thyroglobulin antibody 4.9 IU/mL (0-4), thyroglobulin 159 ng/mL (1.3-31.8), Microsomal Antibody 1096.2 IU/mL (0-9), and Alpha subunit- 3.4 ng/mL (reference range <1.02). A repeat MRI of the pituitary at the time of admission showed an enlarged pituitary, 13x10.1 mm, which appeared slightly improved from the MRI 3 weeks prior to presentation, with decreased involvement of the optic chiasm (Figures 2(a) and 2(b)).

**Figure 3:**
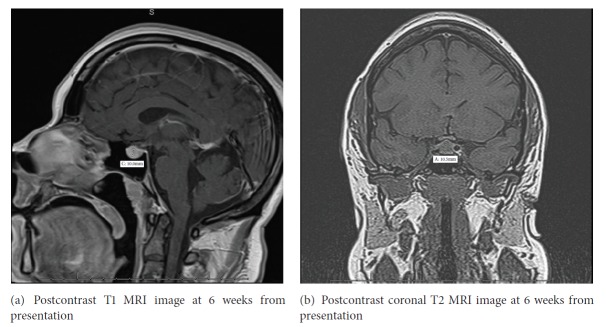


She was initially evaluated for surgery, for presumed diagnoses of pituitary macroadenoma; however prompt recognition of severe primary hypothyroidism causing pituitary hyperplasia prevented any unnecessary surgical intervention. She was started on levothyroxine and liothyronine replacement therapy. Six weeks after her initial presentation, laboratory evaluation showed improvement in thyroid function test with a (Table 1) TSH of 11.23 mIU/mL, FT4- 1.25 ng/dL, Total T3- 95 ng/dL (48-178), free T3 5.7 pg/mL, and Alpha subunit 0.53 ng/mL. A repeat MRI of the pituitary also showed decrease in the height of the pituitary gland to 10.5 mm and with less involvement of the optic chiasm (Figures 3(a) and 3(b)). Within the pituitary gland, there was a 3 mm hypoenhancing focus in the right adenohypophysis suggestive of a microadenoma. She is currently doing well with significant improvement in her symptoms. Repeat laboratory evaluation 3 months after her initial presentation showed complete normalization of thyroid function tests (Table 1) with TSH 0.77 mIU/mL, Total T3 141 ng/dL, and FT4 1.63 ng/dL.

**Figure 4:**
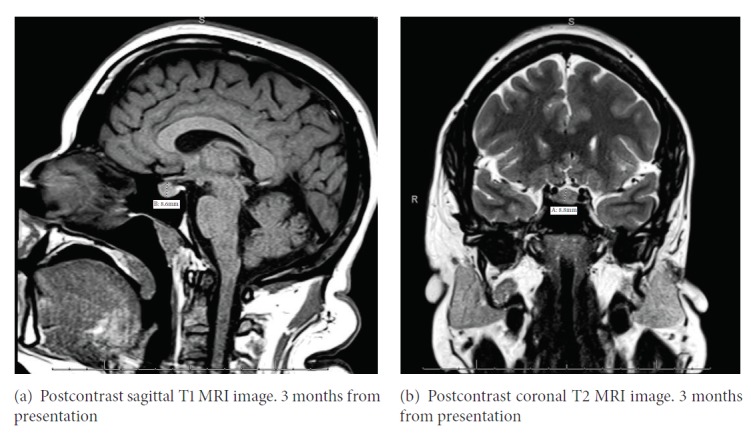


MRI of the pituitary 3 months after presentation shows that the pituitary measures 9.5 mm with a persistent 5 mm hypoenhancing focus in the right adenohypophysis (Figures 4(a) and 4(b)). The rest of the anterior pituitary hormone levels continue to be within reference range. The small microadenoma is thought to be an incidentaloma and is being monitored.

## 3. Discussion

Pituitary hyperplasia in profound and severe primary hypothyroidism was first recognized from the autopsy of a cretin in 1851 [51]. We performed a review of literature of all the case reports, case series, and reviews of adult patients with primary hypothyroidism and pituitary hyperplasia published in the English language (Supplement: Table 2) [2, 6–50]. We excluded case reports, case series, and reviews that only include pediatric cases or were published in a language other than English. The incidence of pituitary hyperplasia in primary hypothyroidism has not been clearly identified, but recent literature suggests that it may be more common than previously accepted. Autopsy studies, by Scheithauer et al. [52] in patients with primary hypothyroidism, have shown diffuse and nodular thyrotropic cell hyperplasia in 69% and 25% of glands, respectively, and a crude correlation was observed between the degree of thyrotropic cell hyperplasia and the relative lack of thyroid hormone replacement therapy. While all patients with pituitary hyperplasia due to primary hypothyroidism will exhibit some signs or symptoms of hypothyroidism, only 38% present with a chief complaint suggestive of hypothyroidism on initial presentation. Additionally up to 25% of the patients with primary hypothyroidism and associated pituitary hyperplasia will present with “tumoral signs” or presence of pituitary enlargement and 36% will present with signs or symptoms of hyperprolactinemia on initial presentation [53]. This is similar to our patient, who although had classic symptoms of primary hypothyroidism, sought medical attention primarily for headache and neurological symptoms.

Prolonged primary hypothyroidism leads to pituitary hyperplasia due to loss of negative feedback from lack of circulating thyroxine (T4) and triiodothyronine (T3), leading to excessive thyrotropin releasing hormone (TRH) secretion from the hypothalamus. Pituitary “tumorous” hyperplasia due to primary hypothyroidism occurs most frequently, accounting for approximately 33.3% of cases of secondary pituitary hyperplasia from end organ insufficiencies [40]. It is interesting to note that thyrotrophs comprise only 5%-10% of the adenohypohysial cells but can still cause significant pituitary enlargement [29, 53]. This can partly be explained by the fact that the number of thyrotrophs can increase substantially to up to 34% of the all adenohypophysial cells in patients with hypothyroidism [53]. This has been confirmed by histologic examination of the pituitary gland in cases where surgery was inadvertently performed [10, 21, 29]. As early as 1976, Yamada et al. [54] suggested that there was pituitary enlargement in patients with primary hypothyroidism. They showed evidence that 81% of patients with hypothyroidism have an abnormal increase in the volume of the sella turcica. In addition, the volume of sella turcica was directly related to the level of TSH and inversely related to the circulating thyroxine levels, suggesting that hyperplasia causes an increase in pituitary size [54].

Other observational studies also show a correlation between serum TSH level and the degree of pituitary enlargement. In an observational study by Khawaja et al. [37] which enrolled patients with TSH greater than 50 *μ*IU/mL, pituitary enlargement on MRI was found in 37 of the 53 patients (70%), with 31 of the 37 patients (84%) having TSH levels of ≥100 *μ*IU/mL. In the same study, younger patients, mostly females, with primary hypothyroidism tended to have higher incidence of pituitary enlargement [37]. A review of the current literature of several pediatric cases and most cases in adults also suggest the same, which is similar to our patient.

Another common theme, in patients with profound primary hypothyroidism and pituitary hyperplasia, is the presence of clinical and/or biochemical hyperprolactinemia. Profoundly hypothyroid patients will have concomitant hyperprolactinemia related to the elevated TRH level. In a study by Honbo et al. [55] in 1978, the serum prolactin level was found to be elevated in 39% of patients with untreated primary hypothyroidism. A significant correlation was found between serum prolactin and TSH, suggesting that the elevated serum prolactin level in primary hypothyroidism is mediated by TRH through negative feedback from hypothyroidism and not primarily by stalk effect [55]. The response of prolactin to TRH is also exaggerated in hypothyroid states vs. euthyroid states and is blunted in hyperthyroid states [56]. This has been highlighted in the literature in several instances, confounding the diagnosis and patients may be mislabeled with a pituitary adenoma or prolactinoma [6, 14–24, 28, 29, 35, 41, 49]. This was also the case in our patient, where she had clinical symptoms of hyperprolactinemia and mild elevation of serum prolactin levels. Moreover, pituitary lactotroph hyperplasia has also been identified on histology in patients with primary hypothyroidism [52].

The presence of pituitary enlargement and elevated TSH levels raises the possibility of a thyrotropin producing pituitary adenoma. However, those tend to be exceedingly rare, accounting for about 0.5% of all pituitary adenomas [53]. It is very important to make an accurate diagnosis to avoid devastating consequences of inappropriate pituitary surgery. The differential diagnosis for our patient included a pituitary adenoma, pituitary hyperplasia, thyroid hormone resistance, or hypophysitis. The presence of biochemical hypothyroidism and response to thyroid hormone replacement made the diagnosis of pituitary hyperplasia in our patient. Biochemical excess or deficiency for other pituitary hormones was ruled out. Appropriate response to thyroid hormone replacement therapy ruled out thyroid hormone resistance. Our patient had a mildly elevated alpha subunit initially which was likely related with profound hypothyroidism and subsequent testing revealed normal levels. In other reported cases, chronic untreated primary hypothyroidism with pituitary hyperplasia has caused long term sequelae, with complete pituitary failure and panhypopituitarism with appearance of empty sella syndrome [7, 44]. Other confounding clinical scenarios have been reported with “pseudoacromegaly,” where patients had clinical signs and symptoms suggestive of acromegaly and imaging suggestive of pituitary adenoma; however a complete clinical history and biochemical evaluation revealed primary hypothyroidism [43, 50].

Although there are certain imaging characteristics that are traditionally associated with either pituitary hyperplasia or an adenoma, even with the use of modern imaging techniques like computed tomography (CT) and magnetic resonance imaging (MRI) with contrast injection, it is still challenging to distinguish between a pituitary adenoma and hyperplasia. The traditional CT criteria for pituitary macroadenoma include homogeneous enlargement of the gland to a height of greater than 10 mm, with or without erosion of the floor of the sella and deviation of the stalk [27]. Although an MRI offers better delineation of adenohypophyseal anatomy, its use cannot always reliably differentiate one from the other. Pituitary hyperplasia is often characterized as a homogeneously enhancing lesion [57] or a midline prominence of a pituitary mass with smooth contours (the “nipple sign”) on a MRI [27]. While MRI findings suggestive of a TSH-secreting adenoma, include a central enhancing mass with a rim of normal compressed pituitary tissue, this can also be seen in pituitary hyperplasia [26, 27].

Appropriate biochemical testing is needed to guide management in such situations. Our case echoes previous case reports, where MRI of the brain was unable to rule out what was considered a symptomatic pituitary adenoma as the cause of pituitary enlargement. As a result, surgical intervention involving removal of the pituitary mass via transphenoidal approach was planned. Surgery was however avoided since thyroid hormone replacement demonstrated regression of the pituitary size on follow-up imaging. This response to therapy suggests TRH-related hyperplasia as the primary cause of pituitary enlargement. It also demonstrates the importance of approaching surgical management of pituitary enlargement in cases of profound primary hypothyroidism with caution. However, it should be noted that increased cell multiplication in cases of hyperplasia may lead to neoplastic transformation of thyrotropin cells and formation of independent TSH producing adenomas, where surgery may be necessary [58]. As a result, early response to hormone replacement therapy does not rule out an underlying neoplasm, and patients should continue to be followed by serial imaging, to track future changes in pituitary size and to evaluate for any adenomas that may be masked by the hyperplasia.

In patients who did undergo pituitary surgery, usually no adenomas were found and histologic examination revealed thyrotrophs that were large, oval with eccentric nucleus, and abundant slightly acidophilic cytoplasm and resemble those seen in the hypothyroid rat pituitary [29]. In addition, in a case series of 3 patients who underwent surgeries for suspected pituitary adenomas, there was combined hyperplasia of both thyrotrophs and lactotrophs. Histologically, no adenomas were identified; however these were suspected clinically, radiologically and in 2 cases, and identified operatively as well [21]. In the previously mentioned autopsy study by Scheithauer et al. [52] on patients with primary hypothyroidism, 94% of the glands showed thyrotroph hyperplasia without significant mass formation and almost 20% demonstrated lactotroph hyperplasia.

It has been shown that pituitary hyperplasia can progress rapidly following the induction of a hypothyroid state. In a series of 14 patients by Shimono et al. [57] who underwent radioactive iodine ablation for distant metastasis for thyroid cancer, the hypothyroid patients had significantly larger pituitary volume both quantitatively and visually on MRI, in comparison to healthy euthyroid volunteers within 3-5 weeks of induction of hypothyroid state. Conversely, replacement with thyroid hormones has also been shown to decrease pituitary size, even as early as 1 week [27]. The rapid response within 1 week was hypothesized by the authors of this study to have been related with the use of high triiodothyronine load used to investigate for thyroid hormone resistance and replacement with high doses of supplemental thyroid hormone [27]. In the study by Shimono et al. [57], the pituitary size decreased 5 weeks after thyroid hormone replacement. Khawaja et al. [37] reported regression of pituitary hyperplasia in 85% of patients who had a follow-up MRI. Most patients will tend to respond anywhere between 4 weeks to 3 months, with documented regression in almost all cases. The same was true for our patient, who had a good response to replacement with thyroid hormone within 6 weeks. The molecular mechanisms underlying this effect of thyroid hormone causing regression of pituitary hyperplasia remains unknown and speculative. However, it may likely be similar to that of dopamine agonist therapy induced reduction in pituitary volume in prolactinomas [59].

## 4. Conclusion

Pituitary hyperplasia in primary hypothyroidism is not uncommon and pituitary enlargement can be frequently observed on imaging. However, imaging with CT or MRI may not be able to reliably differentiate between an adenoma and hyperplasia and biochemical testing should guide diagnosis and management. Both radiographic imaging results and biochemical testing results are essential prior to proceeding with surgical intervention. Patients with hypothyroidism, who have pituitary enlargement incidentally diagnosed on brain imaging for other reasons, should be treated with thyroid hormone replacement and close follow-up and with repeat imaging.

## Figures and Tables

**Table 1:** Thyroid function profile trend.

	TSH mIU/mL	Free T4 (ng/dL)	Free T3 (pg/mL)	Total T3 (ng/dL)
Baseline	251.21	0.44	1.5	-
6 weeks	11.23	1.25	-	95
4 months	0.77	1.63	-	141

## Supplementary Materials

Supplementary Materials
* Supplement: Table 2.* Case reports and series of pituitary hyperplasia secondary to hypothyroidism: year published; age; number of patients; gender; initial TSH, PRL; imaging modality used and pituitary size; visual symptoms; pituitary size; and clinical outcome.
