# Supplementary material for: Pituitary Hyperplasia in Severe Primary Hypothyroidism: A Case Report and Review of the Literature
Source: Case Rep Endocrinol. 2019 Jun 25;2019:2012546. doi: 10.1155/2019/2012546 (PMC6614958; doi:10.1155/2019/2012546)
Supplement: Supplementary Materials — Supplement: Table 2. Case reports and series of pituitary hyperplasia secondary to hypothyroidism: year published; age; number of patients; gender; initial TSH, PRL; imaging modality used and pituitary size; visual symptoms; pituitary size; and clinical outcome. [file 2012546.f1.pdf]

Table 2: Case reports in Adults with Pituitary Hyperplasia and Primary Hypothyroidism											
**Year published**	**Author**	**Number of patients**	**Gender(s)**	**Age**	**Clinical Symptoms**	**Initial TSH**	**Initial PRL**	**Imaging Modality Pituitary Size**	**Visual Deficits**	**Hyperprolactinemia**	**Comments**
1976	Keye et al[6]	1	Female	18	Delayed puberty, growth	971 µIU/mL (ref <5)	1110 µl nq/mL (ref <100)	Pneumoencephalopgra, Brain angiography. Enlarged sella.	No	Yes	Complete resolution
1976	Vagenakis et al[7]	2	Male	71, 64	Lethargy, headaches; lethargy	18 µU/mL; 36 µU/mL	-	X ray and Tomogram. Sella volume 1920 mm^3; 2 cm x 2 cm.	In 1 case	No	Pituitary failure
1978	Jawadi et al[8]	1	Female	20	Amenorrhea, galactorrhea	210 ImU/mL	43-77 ng/mL (ref <15)	Pneumoencephalogram. Enlarged sella.	No	Yes	Complete resolution
1979	Pita et al[9]	1	Female	27	Weight gain, fatigue, cold intolerance	295 µU/mL (ref 0-12)	-	CT Scan. 2 cm x 1.5 cm x 4.5 cm.	No	No	Complete resolution
1980	Katz et al[10]	1	Female	39	Post surgical hypothyroidism. Visual disturbances and bitemporal hemianopsia.	21.4 (no units available)	-	X ray and CT Scan. 2.5 cm x 2.5 cm x 2.5 cm	Yes	No	Surgery
1981	Silver et al[11]	1	Female	23	Oligomenorrhea, galactorrhea	848 µU/mL (ref 0-6)	17 ng/dL (ref 0-20)	CT Scan (no pituitary size available)	No	Yes	Complete resolution
1982	Gup et al[12]	2	Female; Male	29; 64	Oligomenorrhea, galactorrhea; Focal weakness	384 µU/mL; 115 µU/mL (ref <2-6)	27 ng/dL; unknown (ref 6-20)	CT Scan. Enlarged pituitary.	No; Yes	Yes; No	Complete resolution; Surgery

Table 2: Case reports in Adults with Pituitary Hyperplasia and Primary Hypothyroidism

Year published	Author	Number of patients	Gender(s)	Age	Clinical Symptoms	Initial TSH	Initial PRL	Imaging Modality Pituitary Size	Visual Deficits	Hyperprolactinemia	Comments
1983	Valenta et al[13]	2	Female	29; 28	Amenorrhea; Amenorrhea	98 µU/mL; 360 µU/mL (ref <6)	23 ng/dL; 46 ng/dL (ref 6-20)	CT Scan. 1.1 cm; 9 mm.	No	Yes	Complete resolution
1984	Khalil et al[14]	1	Female	32	Oligomenorrhea	350 IU/L (ref <10)	51µg/L (ref 0-30)	CT Scan. Enlarged sella.	No	Yes	Surgery
1984	Groff et al[15]	2	Female	20; 24	Oligomenorrhea, amenorrhea; Galactorrhea	319 µU/mL; 70 µU/mL (ref <8)	84.2 ng/mL; 136.2 ng/mL (ref 4-35)	CT Scan. Intrasellar enhancing mass with suprasellar extension in both cases.	No	Yes	Complete resolution
1985	Bilaniuk et al[16]	1 adult + 2 pediatric	Female	30	Amenorrhea, galactorrhea	700 µU/mL (ref <40)	85 ng/mL (ref 0-30)	CT Scan. 19 mm.	No	Yes	Complete resolution
1986	Heyburn et al[17]	1	Female	26	Amenorrhea	620-960 mU/L (ref <5)	1097-2464 mU/L (ref <420)	CT Scan. 8 mm with suprasellar extension.	No	Yes	Complete resolution
1986	Christopoulos et al[18]	1	Female	42	Galactorrhea, Headache	>45 mU/L	1820 mU/L (ref <360)	CT Scan. Enlarged pituitary.	No	Yes	Complete resolution

Table 2: Case reports in Adults with Pituitary Hyperplasia and Primary Hypothyroidism											
**Year published**	**Author**	**Number of patients**	**Gender(s)**	**Age**	**Clinical Symptoms**	**Initial TSH**	**Initial PRL**	**Imaging Modality Pituitary Size**	**Visual Deficits**	**Hyperprolactinemia**	**Comments**
1987	Grubb et al[19]	2	Female	29; 25	Fatigue, weight gain, cold intolerance, constipation, galactorrhea, amenorrhea; Fatigue	>50 µU/mL; >50 µU/mL (ref 0.2-5.5)	36 ng/mL; 67 ng/mL (ref 3-15)	CT Scan. Enlarged pituitary in both cases.	No	Yes	Complete resolution
1987	Fujii et al[20]	2 (1 patient with hypogonadism)	Female	39; 30	Amenorrhea, galactorrhea; amenorrhea, galactorrhea.	732 µU/mL; 660 µU/mL (ref < 5.7)	96 ng/mL; 73.7 (ref < 18.2)	CT Scan. Enhancing mass with suprasellar extension in all cases.	No	Yes	Complete resolution
1988	Pioro et al[21]	3	2 Female, 1 Male	24/F, 23/F, 44/M	Galactorrhea, amenorrhea; Galactorrhea, amenorrhea, infertility; Impotence	179 µU/mL; 1.9 µU/mL; 233 µU/mL (ref <10)	73 ng/mL; 68 ng/mL; 23 ng/mL (ref 0-30)	Skull X-ray, Angiogram suggests pituitary "microadenoma";<br>Skull Tomogram shows thinning and expansion of sella;<br>CT Scan shows intrasellar mass with suprasellar enlargement.	No	Yes (in 2 cases)	Surgery

Table 2: Case reports in Adults with Pituitary Hyperplasia and Primary Hypothyroidism											
**Year published**	**Author**	**Number of patients**	**Gender(s)**	**Age**	**Clinical Symptoms**	**Initial TSH**	**Initial PRL**	**Imaging Modality Pituitary Size**	**Visual Deficits**	**Hyperprolactinemia**	**Comments**
1989	Ahmed et al[22]	5 (3 adults + 2 pediatric)	Female	19; 27; 39	Primary amenorrhea; Secondary amenorrhea; Secondary amenorrhea	470 mU/L; 439 mU/L; 230 mU/L (ref 1.1-7.2)	69 µg/L; 59 µg/L; 143 µg/L (ref 1.3-20.8)	CT Scan. Suprasellar tumor in all cases.	No	Yes	Complete resolution
1990	Hutchins et al[23]	1	Female	29	Galactorrhea, amenorrhea, cold intolerance, dry skin	450 µIU/mL (ref 0-10)	46.5 µg/L (ref 0-13.8)	MRI. 14 mm.	No	Yes	Complete resolution
1990	Chan et al[24]	3	2 Female, 1 Male	24/F, 33/F, 61/M	Menorrhagia; Amenorrhea, Galactorrhea; Bitemporal hemianopsia	61 mU/L; 9.6 mU/L; 32.6 mU/L (ref 0.5-5)	1750 mU/L; 520 mU/L (ref <520); 661 mU/L (ref <290)	CT Scan. Pituitary enlargement with suprasellar extension.	Yes in 1 case	Yes	Surgery in one case; complete resolution in 2 cases
1992	Fulcher et al[25]	1	Male	23	Short stature, fatigue	586 µIU/mL (ref <4)	-	MRI. Enlarged pituitary.	Yes	No	Complete resolution
1996	Wolansky et al[26]	1	Female	27	Amenorrhea, galactorrhea	283 µU/mL (ref 0-3)	43 ng/mL (ref 0-20)	CT Scan, MRI. 17 mm.	No	Yes	Complete resolution
1997	Sarlis et al[27]	1	Female	26	Post radioactive iodine ablation	563.3 mU/L (ref 0.4-4.4)	-	MRI. Symmetric enlargement 12 mm in height.	No	No	Complete resolution

Table 2: Case reports in Adults with Pituitary Hyperplasia and Primary Hypothyroidism											
Year published	Author	Number of patients	Gender(s)	Age	Clinical Symptoms	Initial TSH	Initial PRL	Imaging Modality Pituitary Size	Visual Deficits	Hyperprolactinemia	Comments
1997	Ozbey et al[28]	1	Female	32	Amenorrhea, galactorrhea	97 mU/mL (ref 04.-4)	77 µg/mL (ref 2-20)	MRI. 17 mm x 15 mm.	No	Yes	Complete resolution
1999	Alkhani et al[29]	1	Female	51	Headache, sensory loss, motor symptoms	>100 mU/L (ref 0.4-5.5)	90 ng/L (ref 0-25)	MRI. 1.6 cm x 1.2 cm x 1 cm homogeneously enhancing mass.	No	Yes	Surgery
1999	Young et al[30]	3 (2 pediatric+ 1 adult)	Female	25	Galactorrhea, Syncope	881. mU/L (ref 0.4-6)	48.3 ng/mL (ref 3-24)	MRI. Enlarged pituitary.	No	Yes	Biopsy, complete resolution
1999	Yamagishi et al[31]	1	Female	18	Malaise	431.7 µU/mL (ref 0.4-5)	-	MRI. 2 cm x 2 cm x 1 cm homogeneously enhancing mass with suprasellar extension.	No	No	Complete resolution
2000	Nicholas et al[32]	1	Female	33	Fatigue, amenorrhea, galactorrhea	677 mcu/mL (ref 0.4-5.5)	114 ng/mL (ref 1.3-24.2)	MRI. Pituitary enlargement.	No	Yes	Complete resolution
2000	Manocha et al[33]	1	Male	22	Headache	150 µU/L	-	CT Scan 1.1 cm x 1.1 cm x 1.2 cm.	No	No	Complete resolution
2003	Hoogenberg et al[34]	1	Male	51	Hypothyroidism	120 mU/L	580 mU/L (ref 50-200)	MRI. 12 mm with normal gadolinium distribution.	-	Yes	Complete resolution

Table 2: Case reports in Adults with Pituitary Hyperplasia and Primary Hypothyroidism

Year published	Author	Number of patients	Gender(s)	Age	Clinical Symptoms	Initial TSH	Initial PRL	Imaging Modality Pituitary Size	Visual Deficits	Hyperprolactinemia	Comments
2004	Kroese et al[35]	1	Female	36	Amenorrhea, galactorrhea	>75 mU/L (ref 0.4-4)	103 g/L (ref 0-20)	MRI. 14 mm x 20 mm x 12 mm with suprasellar extension.	No	Yes	Complete resolution
2005	Joshi et al[36]	1	Female	28	Medication non-compliance	177 mIU/ml	-	MRI. 1.2 cm with suprasellar extension.	No	No	Complete resolution
2006	Khawaja et al[37]	28 (9 pediatric)	25 Females, 3 males	19-65	Variable	50-4191 μIU/mL	7-100 ng/mL	MRI. 312 mm^3 - 1567 mm^3.	Yes in 2 patients.	Yes (in some pediatric cases)	Resolution in most cases.
2011	Passeri et al[38]	1	Female	23	Fatigue, Polymenorrhea	1578 μU/mL	113 ng/mL	MRI. 23 mm x 23 mm x 10 mm sellar and suprasellar mass.	No	Yes	Complete resolution
2011	Agrawal et al[39]	1	Female	22	Headache, fatigue	150 μU/dL (ref 0.3-5.5)	35 IU/dL	CT Scan, MRI. Diffuse enlargement.	No	No	No follow up imaging
2012	Han et al[40]	15	6 Males, 9 Females	13-46	Variable	85-190 μIU/mL (ref 0.38-4.34)	50.46-180.75 ng/mL (ref 3.34-26.72)	CT Scan, MRI. Variable size.	No	Yes	Complete resolution
2012	Rajput et al[41]	1	Female	32	Amenorrhea	660.18 mIU/ml (ref 0.35–5.5)	101.8 ng/ml (ref 3.1–25)	MRI. 19 mm x 12.5 mm x 12 mm.	No	Yes	Complete resolution

Table 2: Case reports in Adults with Pituitary Hyperplasia and Primary Hypothyroidism											
Year published	Author	Number of patients	Gender(s)	Age	Clinical Symptoms	Initial TSH	Initial PRL	Imaging Modality Pituitary Size	Visual Deficits	Hyperprolactinemia	Comments
2012	Eiland et al[42]	1	Female	23	Cold intolerance, fatigue	1398 lIU/mL (ref 0.34–5.6)	1260.8pM/L (ref 43.5–1043.5)	MRI. Enlarged pituitary with homogenous enhancement.	Yes	No	Complete resolution
2012	Kumar et al[43]	1	Male	22	Acromegaly	122.2 mIU/l (normal 0.3–4.5)	24 ng/ml (ref 0–15)	MRI. 1.6 cm x 1.1 cm pituitary mass with suprasellar extension.	No	No	Complete resolution
2012	Dutta et al[44]	1	Male	27	Short stature, fatigue	>50 μU/mL	-	CT Scan, MRI. Empty sella.	No	No	Pituitary failure
2015	Neves et al[45]	1	Female	21	Amenorrhea, galactorrhea	>500 mU/L (0.4–4.3)	1000 mIU/L (0-700)	MRI. Homogenously enhancing 19 mm x 17 mm x 12 mm mass with suprasellar and parasellar extension.	No	Yes	Complete resolution
2015	De Sousa et al[2]	4 (1 with hypothyroidism)	Female	19	Abdominal pain	566 mIU/L (ref 0.4-3.5)	782 mIU/L (85-500)	MRI. 13 mm with suprasellar extension.	No	Yes	Complete resolution
2015	Moumen et al[46]	1	Male	35	Headache	>100 μIU/mL (ref 0.34-5.6)	22.5 ng/mL (ref 2.64-13.13)	MRI. 12.5 mm.	Yes	No	Complete resolution

Table 2: Case reports in Adults with Pituitary Hyperplasia and Primary Hypothyroidism											
Year published	Author	Number of patients	Gender(s)	Age	Clinical Symptoms	Initial TSH	Initial PRL	Imaging Modality Pituitary Size	Visual Deficits	Hyperprolactinemia	Comments
2015	Siddiqi et al[47]	1	Female	18	Decreased visual acuity	>100 µIU/mL (ref 0.27-4.2)	1134 mIU/mL (ref 86-324)	MRI. Sellar mass with suprasellar extension.	Yes	Yes	Complete resolution
2016	Pappy et al[48]	1	Female	63	Abnormal MRI	147 µIU/mL (ref 0.4-4.5)	-	MRI. 5 mm hypodense lesion.	No	No	Complete resolution
2016	Ansari et al[49]	1	Female	67	Loss of consciousness	863.3 mIU/L (ref 0.2-4.2)	3234 mIU/L (ref 102-496)	MRI. 1.9 cm x 1.6 cm x 1.8 cm.	No	No	Complete resolution
2017	Chakraborty et al[50]	1	Male	64	Acromegaly	>75 µIU/mL	33.4 ng/mL	MRI. Enlarged pituitary.	No	No	Complete resolution

Legend: TSH - Thyroid stimulating hormone; PRL - Prolactin; CT - Computed tomography; MRI - Magnetic resonance imaging
